# Net water uptake and ASPECTS in predicting futile recanalization for acute large vessel occlusion stroke: insights from time window stratification

**DOI:** 10.3389/fneur.2026.1741637

**Published:** 2026-02-04

**Authors:** Xiang Li, Qiuxia Xiong, Wenbing Zeng, Yun Wen, Xinghua Liu, Yongmei Li

**Affiliations:** 1 Department of Radiology, The First Affiliated Hospital of Chongqing Medical University, Chongqing, China; 2 Department of Radiology, Chongqing University Three Gorges Hospital, Chongqing, China

**Keywords:** acute ischemic stroke, brain edema, net water uptake, nomogram, prognosis

## Abstract

**Objective:**

This study aimed to evaluate the prognostic value of Alberta Stroke Program Early CT Score (ASPECTS)-based net water uptake (NWU) in predicting futile recanalization (FR) and develop a nomogram integrating NWU and clinical parameters for personalized risk stratification in early or late time windows in acute ischemic stroke (AIS) at admission.

**Methods:**

This retrospective study investigated AIS patients with large vessel occlusion who achieved successful recanalization from January 2022 to November 2024. Baseline ASPECTS-NWU was automatically quantified from the admission CT. The primary outcome was FR, defined as a modified Rankin Scale (mRS) score of 3–6 at 90 days following successful recanalization (modified Thrombolysis in Cerebral Infarction score, 2b/3). Intergroup comparisons of clinical and neuroimaging parameters were performed using the Mann–Whitney U test for continuous variables and the χ^2^ test for categorical measures. Bivariate correlations between NWU values and other covariates were assessed using Spearman’s rho coefficients. Multivariable logistic regression identified independent predictors of FR, and a subsequent nomogram was constructed. The logistic regression model was tested using the 5-fold cross-validation. The predictive accuracy of different models was evaluated using the receiver operating characteristic curve and DeLong’s test.

**Results:**

A total of 168 patients were included, and FR occurred in 95 patients. Patients with FR were older, had higher National Institutes of Health Stroke Scale scores, an increased number of passes, lower ASPECTS, elevated NWU, larger cerebral blood flow (CBF) < 30% volume, and increased hypoperfusion intensity ratio (HIR), regardless of the time window. In the late time window, multivariable analysis identified baseline NWU and ASPECTS as independent predictors of FR. A nomogram integrating these two factors demonstrated strong predictive power, with an area under the curve of 0.880.

**Conclusion:**

Baseline NWU and ASPECTS were independent predictors of AIS patients with FR in the late time window. The developed nomogram integrating NWU with ASPECTS provides a clinically actionable tool for pre-endovascular therapy risk stratification.

## Introduction

Endovascular therapy (EVT) has become the first-line treatment for acute ischemic stroke (AIS) caused by large vessel occlusion (LVO), with timely recanalization significantly improving patient outcome (1). A recent study indicated that the benefit of successful recanalization is largely mediated by limiting infarct growth and reducing edema (2). However, successful recanalization of LVO does not always translate to functional recovery. In real-world clinical practice, approximately 50% of patients still experience poor functional outcomes at 90 days, a phenomenon known as futile recanalization (FR) (3, 4). Growing evidence suggests that microcirculatory dysfunction and the resulting cytotoxic edema play a pivotal role in this discrepancy, as persistent capillary-level ischemia can persist despite proximal vessel recanalization, exacerbating injury through blood–brain barrier disruption and neurovascular uncoupling (5, 6). Therefore, accurately and early assessing the degree of cerebral edema is of substantial value for treatment decision-making and predicting prognosis.

Currently, cerebral edema is commonly assessed using non-contrast computed tomography (NCCT). The Alberta Stroke Program Early CT Score (ASPECTS) provides a regional evaluation of early ischemic changes but offers only a semi-quantitative, binary assessment that cannot capture gradations in hypoattenuation severity (7, 8). Midline shift (MLS) quantifies mass effect as an indirect marker of edema, yet it is a late sign that emerges only after intracranial compensatory reserves are exhausted, limiting its utility in early decision-making (9–11). There remains a need for a more objective, quantitative, and early biomarker of cerebral edema to better predict post-EVT outcomes.

Net water uptake (NWU) has emerged as a promising quantitative neuroimaging biomarker, measuring the extent of hypoattenuation to directly estimate pathological water accumulation in ischemic tissue (12, 13). Although NWU has not yet been used as an imaging indicator for selecting EVT candidates, an increasing number of studies have shown that elevated NWU in ischemic lesions during the acute phase was significantly correlated with adverse clinical sequelae, including malignant edema, progression to malignant infarction, increased risk of hemorrhagic transformation, and unfavorable functional outcomes (14–17). Notably, NWU can be derived directly from NCCT, an imaging modality that is readily available even in primary stroke centers. However, cerebral edema progression is time-sensitive, and whether the predictive value of NWU differs between early and late time windows remains poorly understood.

We hypothesize that baseline ASPECTS-based NWU differ significantly between early and late time windows in LVO-AIS patients and may serve as a pre-EVT biomarker for predicting FR. This study aimed to evaluate the association between NWU and FR across different treatment windows and to develop an integrated predictive nomogram for FR using readily available admission parameters.

## Materials and methods

### Study design and population

This retrospective study was reviewed and approved by the local institutional review board. Written informed consent was waived due to its retrospective design. From January 2022 to November 2024, we retrospectively reviewed consecutive patients with AIS who underwent EVT in our stroke center. The inclusion criteria were as follows: (1) adult patients aged 18 years or older; (2) patients underwent a standardized multimodal stroke CT imaging protocol upon admission, including NCCT, CT angiography (CTA), and CT perfusion (CTP); (3) AIS due to LVO of the internal carotid artery (ICA) and/or the M1 segment of the middle cerebral artery (MCA); (4) stroke onset to admission or last seen well to admission time within 24 h. The exclusion criteria were as follows: (1) pre-stroke modified Rankin Scale (mRS) score > 2; (2) previous intracranial hemorrhage, brain surgery, or large territorial lesion; (3) ischemic lesions in the territory of an anterior cerebral artery or posterior circulation; (4) segmentation error of ASPECTS software; (5) incomplete clinical information; (6) 90-day mRS follow-up data were unavailable; (7) modified Thrombolysis in Cerebral Infarction (mTICI) ≤ 2a; (8) ASPECTS = 10.

### Clinical information

The clinical information was obtained from the electronic medical records, including demographics (age, sex), vascular risk factors (hypertension, diabetes mellitus, atrial fibrillation, coronary artery disease, smoking), admission medications (prior antiplatelet or anticoagulant use), time metrics, pre-stroke mRS, admission mRS, National Institutes of Health Stroke Scale (NIHSS) at admission, blood glucose, neutrophil count, and lymphocyte count. The neutrophil-to-lymphocyte ratio (NLR) was determined as the ratio of neutrophil count to lymphocyte count.

### Image acquisition and protocols

All AIS patients underwent a standardized multimodal stroke imaging on a 256-row CT scanner (Revolution; GE Healthcare, USA) following the order: NCCT, CTP, and CTA. The protocols were as follows: (1) NCCT: tube voltage 100 kVp, tube current 225 mAs, slice thickness 5.0 mm, and scanning ranged from the skull base to the vertex; (2) CTP: tube voltage 80 kVp, tube current 150 mAs, slice thickness 5.0 mm, and scanning ranged from the skull base to the vertex. Twenty-four consecutive axial scans of the brain (Z-axis 160 mm, and 2.0 s temporal resolution) were acquired after injecting 50 mL contrast medium (iopromide, Ultravist 370, Bayer Schering Pharma) at a flow rate of 5 mL/s followed by 50 mL saline chaser; (3) CTA was performed from the aortic arch to the vertex after CTP was completed by injecting 50 mL contrast medium (iopromide, Ultravist 370, Bayer Schering Pharma) at a flow rate of 5 mL/s followed by 50 mL saline chaser, tube voltage 100 kVp, tube current 250 mAs, slice thickness 0.625 mm, pitch value 0.992.

### Image analysis

#### Automated ASPECTS and ASPECTS-NWU quantification

The ASPECTS measurements were automatically calculated using the stroke software tool (RAPID ASPECTS, iSchemaView). The mean Hounsfield unit (HU) within each ASPECTS region was systematically measured. Regions were classified as normal or pathological via a machine learning algorithm, and ischemic involvement was visually mapped in red. A senior neuroradiologist (with 13 years of neuroradiology experience) independently validated all identified ASPECTS territories to ensure segmentation accuracy and anatomical localization reliability. To quantify ischemic changes, the relative HU difference (%) between affected regions (ischemic hemisphere) and their mirroring unaffected areas (contralateral hemisphere) was calculated using the formula:


$$\text{ASPECTS}-\mathrm{NWU}(\%)=[1-(\frac{\text{HUischemic}}{\text{HUnormol}})]\times 100\%$$

### CTP analysis

All source CTP datasets underwent standardized processing with RAPID (iSchemaView), a dedicated perfusion analysis platform. Ischemic core quantification applied a relative cerebral blood flow (CBF) threshold <30% of contralateral reference values, while hypoperfusion assessment utilized time-to-maximum (Tmax) delays exceeding 6 s as volumetric parameters. Hypoperfusion intensity ratio (HIR) was defined by dividing the volumetric measurement of Tmax >10s regions by the corresponding Tmax >6 s volume.

### Evaluation of recanalization

The selection of EVT modalities (stent retriever devices, aspiration systems, or hybrid approaches) with adjunctive strategies, including optional use of balloon-guiding catheters, was determined by the neuro-interventional physician based on real-time angiographic findings and institutional protocols. The degree of recanalization in patients undergoing EVT was assessed by the operator, using the mTICI scale, based on digital subtraction angiography performed after the procedure. Successful recanalization was defined as an mTICI of 2b/3.

### Outcomes

The mRS scores at 90 days were evaluated by a trained nurse through structured telephonic interviews or face-to-face evaluations. FR was defined as mRS 3–6 at 90 days following successful recanalization (mTICI, 2b/3), while non-FR was defined as mRS 0–2 (18).

### Statistical analysis

The Shapiro–Wilk test was employed to determine if the data followed a normal distribution. Continuous data with a normal distribution are reported as the means ± SD, the independent sample test was used for comparison, whereas non-normally distributed data are expressed as median and interquartile range (IQR), the Mann–Whitney U test was used for comparison. Chi-square test or Fisher’s exact test was used for categorical variables comparison.

We evaluated potential multicollinearity among covariates by establishing a variance inflation factor threshold of < 5 as the diagnostic criterion for the absence of significant collinearity. In logistic regressions, the 90-day mRS was modeled as a binary variable (0–2 vs. 3–6). All baseline characteristics associated with mRS score at 90-day in univariable analysis with significant *p* values (*p* < 0.1) were entered into the multivariable logistic regression models to investigate the predictive validity of each parameter for functional outcomes. All patients were divided into two subgroups (early time window, <6 h versus late time window, 6-24 h) based on the stroke onset to admission or last seen well to admission time. In the subgroup analysis, the same logistic regression analysis method was used. Internal validation was performed using a five-fold cross-validation method for the established model. Receiver operating characteristic (ROC) curve analysis was used to assess the performance of different models for predicting FR. The Youden index was utilized to determine the optimal cutoff point, and the sensitivity and specificity were calculated. DeLong’s test was used for ROC curve comparison. Spearman’s correlation coefficient was calculated to assess the correlation between ASPECTS-NWU and ASPECTS, CTP parameters, clinical characteristics, and 90-day mRS. A *p* value < 0.05 was considered significant.

Statistical analyses were conducted using SPSS 26.0 (IBM Corp, Armonk, NY, USA) and the uAI Research Portal (United Imaging, Shanghai, China).

## Results

### Patient characteristics

**Figure 1:**
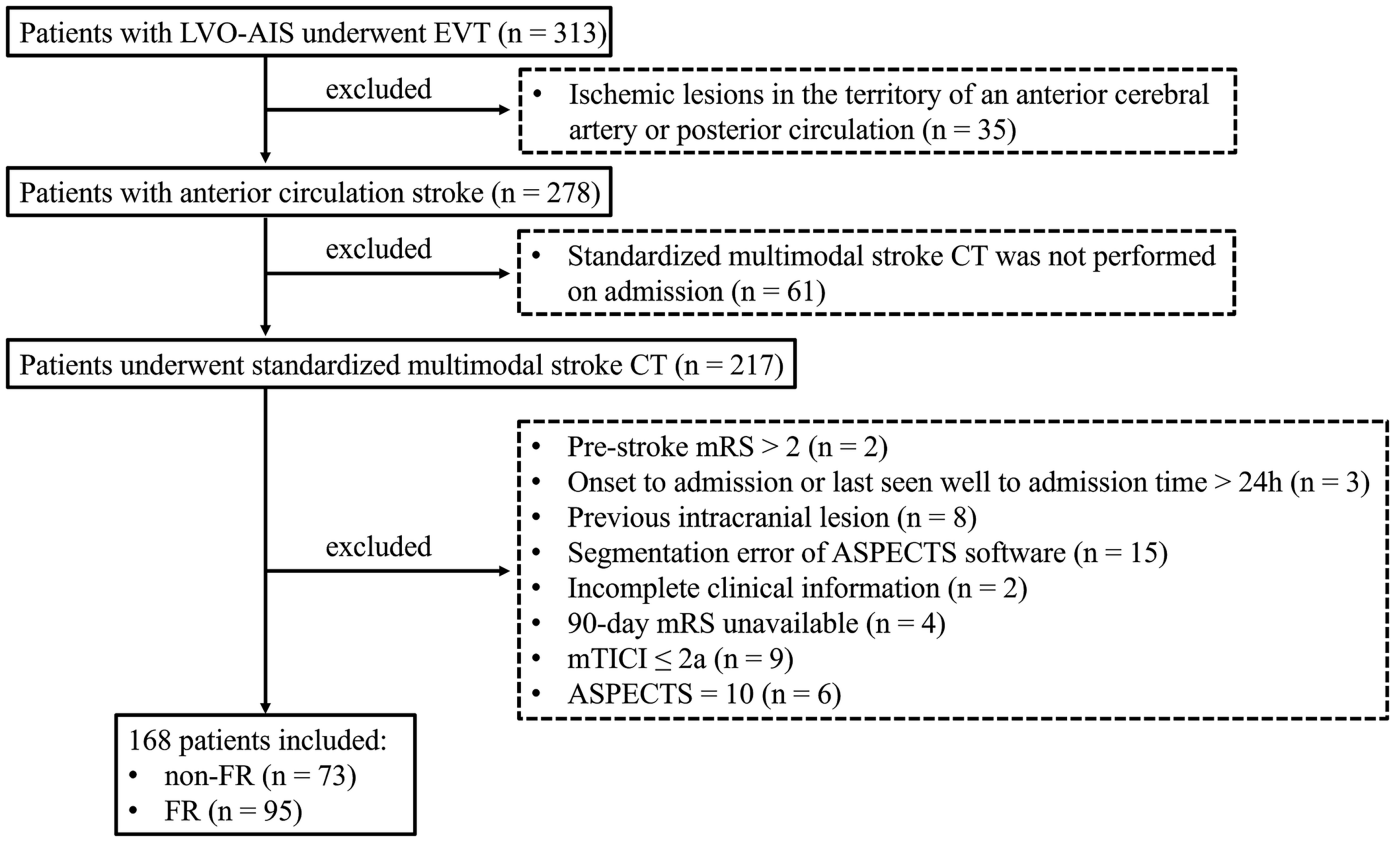
Flowchart of patient selection.

A total of 168 LVO-AIS patients (male, 101; median age, 69.5 years) who underwent EVT and achieved successful recanalization were involved in this study. At 90 days, FR was observed in 95 (56.55%) patients (Figure 1). As listed in Table 1, the median NLR was 4.50 (IQR, 2.85–8.61), the median admission blood glucose was 6.70 (IQR, 5.63–8.28) mmol/L, the median NIHSS score was 12.50 (IQR, 9.00–17.00), and the onset to admission time was 270 (IQR, 180–540) minutes. In the full cohort, the prevalence of vascular risk factors exhibited a heterogeneous distribution, ranging from 13.1 to 46.4%. Automated quantitative NCCT analysis, the median baseline ASPECTS was 6 (IQR, 3–8), and the median baseline NWU% was 6.77% (IQR, 4.84–9.36). On CTP parameters, the median volume of ischemic core (CBF < 30%) and hypoperfusion (Tmax > 6 s) was 16.50 mL (IQR, 0.00–61.00) and 149.00 mL (IQR, 86.50–222.25), respectively. Due to collinearity between Tmax > 6 s and Tmax > 10s, Tmax >10s was used exclusively for calculating the HIR. The median HIR was 0.41 (IQR, 0.14, 0.60).

**Table 1:** Patient characteristics stratified by FR.

Variables	Study cohort, *N* = 168	Non-FR, *N* = 73	FR, *N* = 95	*P*
Age, median (IQR)	69.50 (60.25, 77.00)	67.00 (56.00, 72.00)	74.00 (65.00, 79.00)	< 0.001
Male, *n* (%)	101 (60.12%)	52 (71.23%)	49 (51.58%)	0.011
NLR, median (IQR)	4.50 (2.85, 8.61)	3.80 (2.45, 6.22)	5.26 (2.93, 10.08)	0.010
Admission blood glucose (mmol/L)	6.70 (5.63, 8.28)	6.70 (5.70, 8.02)	6.80 (5.50, 8.80)	0.587
Onset to admission time (minutes)	270.00 (180.00, 540.00)	300.00 (120.00, 600.00)	270.00 (180.00, 480.00)	0.977
Onset to imaging (minutes)	223.50 (127.00, 458.00)	213.00 (97.50, 536.50)	235.00 (141.00, 454.00)	0.462
Onset to puncture (minutes)	388.50 (236.00, 713.00)	382.00 (230.50, 790.50)	411.00 (236.00, 639.00)	0.935
Onset to recanalization (minutes)	523.00 (374.75, 861.75)	505.00 (304.50, 976.50)	543.00 (408.00, 816.00)	0.449
Number of passes	2 (1, 3)	1 (1, 2)	2 (1, 3)	< 0.001
Previous mRS	0 (0,0)	0 (0.0)	0 (0.0)	0.625
Admission mRS	4 (4, 5)	4 (4, 5)	5 (4, 5)	0.002
Admission NIHSS	12.50 (9.00, 17.00)	10.00 (8.00, 15.00)	15.00 (10.00, 18.00)	< 0.001
Baseline ASPECTS	6 (3, 8)	8 (6, 9)	6 (4, 8)	< 0.001
Baseline NWU (%)	6.77 (4.84, 9.36)	5.57 (4.10, 7.72)	8.23 (5.65, 10.78)	< 0.001
Tmax > 6 s(mL)	149.00 (86.50, 222.25)	126.00 (69.00, 182.50)	173.00 (106.00, 239.00)	0.004
CBF < 30%(mL)	16.50 (0.00, 61.00)	0.00 (0.00, 18.50)	34.00 (12.00, 107.00)	< 0.001
HIR	0.41 (0.14, 0.60)	0.25 (0.04, 0.45)	0.48 (0.28, 0.67)	< 0.001
Hypertension, *n* (%)	78 (46.4)	33 (45.2)	45 (47.4)	0.781
Diabetes mellitus, *n* (%)	22 (13.1)	7 (9.6)	15 (15.8)	0.238
CAD, *n* (%)	33 (19.6)	15 (20.5)	18 (18.9)	0.796
Atrial fibrillation, *n* (%)	41 (24.4)	17 (23.3)	24 (25.2)	0.768
Smoking, *n* (%)	60 (35.7)	30 (41.1)	30 (31.6)	0.202
Prior anticoagulant, *n* (%)	23 (13.7)	11 (15.1)	12 (12.6)	0.649
Prior antiplatelet, *n* (%)	16 (9.5)	7 (9.6)	9 (9.5)	0.980

FR, futile recanalization; NLR, Neutrophil-to-Lymphocyte Ratio; mRS, modified Rankin Scale; NIHSS, National Institutes of Health Stroke Scale; ASPECTS, Alberta Stroke Program Early CT Score; NWU, net water uptake; Tmax, time to maximum; CBF, cerebral blood flow; HIR, hypoperfusion intensity ratio; CAD, coronary artery disease.

Patients with FR were significantly older (median age, 74 vs. 67 years, *p* < 0.001), less likely to be men (51.58% vs. 71.23%, *p* = 0.011), had higher NLR (median, 5.26 vs. 3.80, *p* = 0.010), had higher admission mRS (median, 5 vs. 4, *p* = 0.002), and exhibited greater stroke severity (median NIHSS, 15 vs. 10, *p* < 0.001) compared to the non-FR group. Imaging parameters also differed significantly, the FR group demonstrated larger hypoperfusion volumes (Tmax > 6 s: median 173 mL vs. 126 mL, *p* = 0.004), expanded ischemic core (CBF < 30%: median 34 mL vs. 0 mL, *p* < 0.001), increased HIR values (median, 0.48 vs. 0.25, *p* < 0.001), and elevated NWU% (median, 8.23% vs. 5.57%, *p* < 0.001). In terms of perioperative indicators, the number of passes in the FR group was higher than that in the non-FR group (IQR, 2 vs. 1), while the other differences were not statistically significant. Conversely, baseline ASPECTS (median, 6 vs. 8, *p* < 0.001) was significantly lower in the FR group. Additionally, no significant differences were observed in admission blood glucose (*p* = 0.587), onset-to-admission time (*p* = 0.985), or comorbidities such as hypertension (*p* = 0.781) and diabetes (*p* = 0.238).

### Patient characteristics in subgroup

According to the onset to admission time, 104 patients were assigned to the early time window group, and 64 patients were assigned to the late time window group. Patients with FR demonstrated significantly older age, higher NIHSS scores, an increased number of passes, and worse neuroimaging profiles, which included lower ASPECTS, elevated NWU%, larger CBF < 30% volumes, and increased HIR values, regardless of the time window (all *p* < 0.05). More details are presented in Table 2.

**Table 2:** Patient characteristics stratified by time window.

Variables	Early time window (< 6 h), *N* = 104			Late time window (≥ 6 h), *N* = 64		
	Non-FR, *N* = 46	FR, *N* = 58	*P*	Non-FR, *N* = 27	FR, *N* = 37	*P*
Age, median (IQR)	67.00 (58.25, 72.25)	75.00 (65.00, 81.00)	0.002	63.74 ± 12.71	69.62 ± 9.05	0.034
Male, *n* (%)	28 (60.9)	28 (48.3)	0.201	24 (88.9)	21 (56.8)	0.005
NLR, median (IQR)	3.79 (2.20, 6.21)	4.23 (2.47, 9.46)	0.292	3.98 (2.87, 6.22)	7.96 (4.08, 14.09)	0.005
Admission blood glucose (mmol/L)	6.35 (5.70, 7.60)	6.85 (5.70, 8.08)	0.278	7.24 (5.60, 8.70)	6.70 (5.15, 10.25)	0.989
Onset to admission time (minutes)	180.00 (120.00, 240.00)	180.00 (120.00, 240.00)	0.621	780.00 (600.00, 1440.00)	600.00 (480.00, 1020.00)	0.068
Onset to imaging (minutes)	148.50 (77.75, 213.00)	152.00 (103.75, 222.00)	0.366	692.00 (413.00, 1112.00)	492.00 (418.50, 907.00)	0.484
Onset to puncture (minutes)	266.00 (168.75, 339.00)	275.00 (216.75, 375.25)	0.421	1110.00 (714.00, 1536.00)	668.00 (567.00, 1222.00)	0.027
Onset to recanalization (minutes)	376.50 (276.50, 475.25)	423.00 (339.75, 513.75)	0.051	1210.00 (855.00, 1641.00)	840.00 (686.00, 1372.00)	0.072
Number of passes	1 (1, 2)	2 (1, 3)	0.010	2 (1, 2)	2 (1, 3)	0.011
Previous mRS	0 (0, 0)	0 (0, 0)	0.390	0 (0, 0)	0 (0, 0)	0.746
Admission mRS	4 (4, 5)	5 (4, 5)	0.001	4 (4, 5)	4 (4, 5)	0.277
Admission NIHSS	12.07 ± 6.10	15.19 ± 5.84	0.009	9.78 ± 5.63	13.27 ± 5.72	0.018
Baseline ASPECTS	7.0 (5.8, 8.0)	4.5 (2.0, 6.0)	< 0.001	8.0 (7.0, 9.0)	4 (2.5, 6.0)	< 0.001
Baseline NWU%	5.83 ± 2.59	7.31 ± 3.47	0.017	5.57 (3.48, 7.83)	10.54 (7.05, 13.63)	< 0.001
Tmax > 6 s(mL)	127.00 (76.00, 152.25)	176.00 (100.00, 274.25)	0.005	125.00 (68.00, 233.00)	168.00 (109.50, 217.50)	0.407
CBF < 30%(mL)	4.50 (0.00, 23.25)	46.50 (14.50, 136.25)	< 0.001	0.00 (0.00, 10.00)	19.00 (9.00, 65.50)	< 0.001
HIR	0.35 (0.04, 0.49)	0.51 (0.33, 0.71)	0.001	0.14 (0.03, 0.36)	0.47 (0.21, 0.62)	< 0.001
Hypertension, *n* (%)	22 (47.8)	26 (44.8)	0.761	11 (40.7)	19 (51.4)	0.401
Diabetes mellitus, *n* (%)	4 (8.7)	7 (8.6)	1.000	3 (11.1)	10 (27.0)	0.118
CAD, *n* (%)	12 (26.1)	15 (25.7)	0.979	3 (11.1)	3 (8.1)	0.691
Atrial fibrillation, *n* (%)	16 (34.8)	20 (34.5)	0.975	1 (3.7)	4 (10.8)	0.387
Smoking, *n* (%)	17 (37.0)	16 (27.6)	0.308	13 (48.1)	14 (37.8)	0.409
Prior anticoagulant, *n* (%)	9 (19.6)	11 (19.0)	0.939	2 (7.4)	1 (2.9)	0.568
Prior antiplatelet, *n* (%)	5 (10.9)	7 (12.1)	0.849	2 (7.4)	2 (5.9)	1.000

FR, futile recanalization; NLR, Neutrophil-to-Lymphocyte Ratio; mRS, modified Rankin Scale; NIHSS, National Institutes of Health Stroke Scale; ASPECTS, Alberta Stroke Program Early CT Score; NWU, net water uptake; Tmax, time to maximum; CBF, cerebral blood flow; HIR, hypoperfusion intensity ratio; CAD, coronary artery disease.

### Association between NWU and the other variables

**Figure 2:**
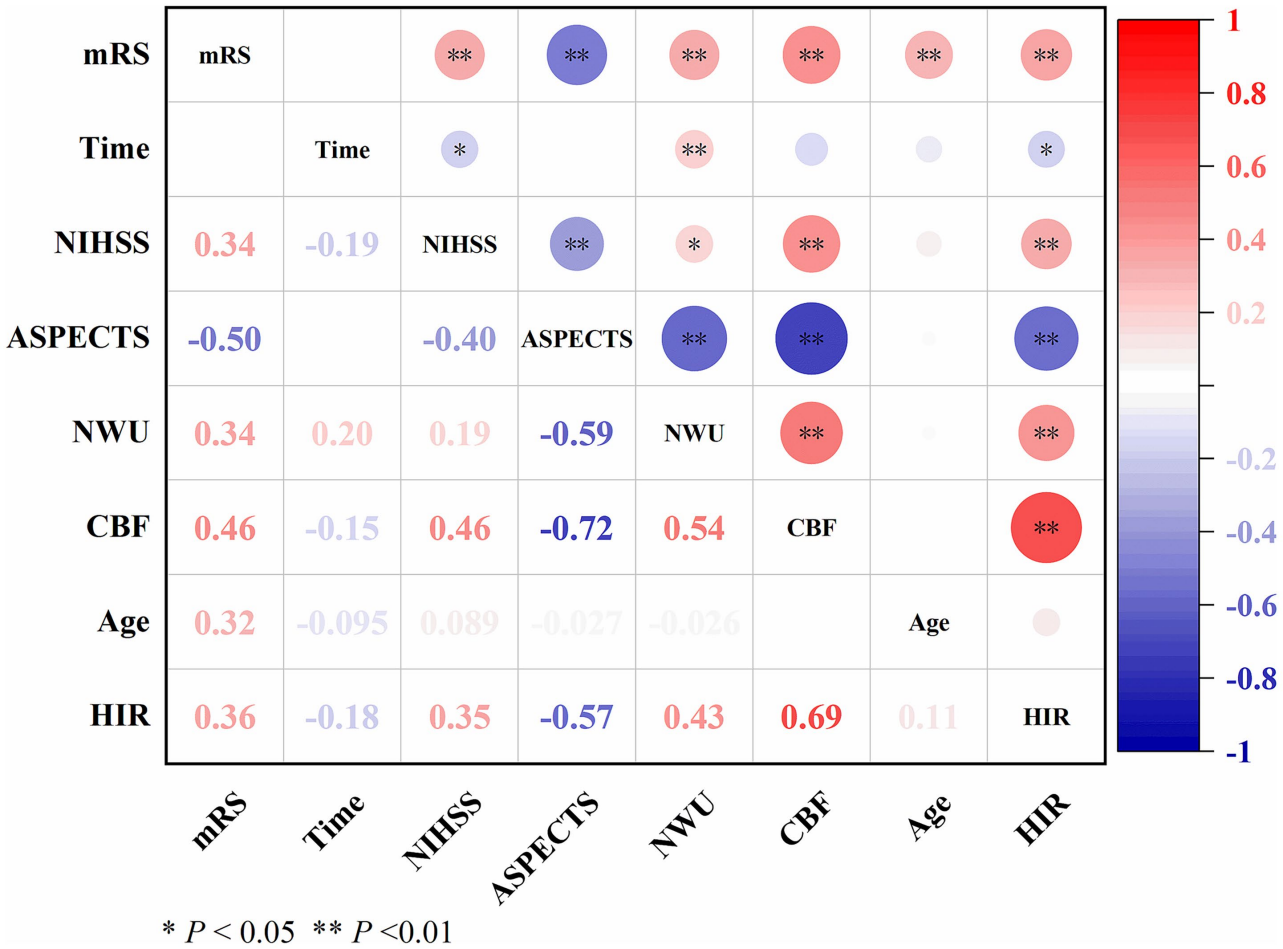
Correlation between NWU and other variables.

As shown in Figure 2, NWU demonstrated a moderate positive correlation with CBF (*ρ =* 0.54, *p* < 0.01) and HIR (*ρ =* 0.43, *p* < 0.01), weak positive correlations with onset time (*ρ* = 0.20, *p* < 0.01), NIHSS (*ρ* = 0.19, *p* < 0.05) and mRS (*ρ* = 0.34, *p* < 0.01), and moderate negative correlation with ASPECTS (*ρ* = −0.59, *p* < 0.01).

### Independent predictors of FR in full cohort and subgroups

In the full cohort, an increasing age (aOR, 1.078, 95% CI: 1.033–1.124; *p* < 0.001) and a higher baseline ASPECTS (aOR, 0.711, 95% CI: 0.555–0.907; *p* = 0.006) were independently associated with FR in Model A. After incorporating perioperative variables in Model B, both age (aOR, 1.091; 95% CI, 1.042–1.143; *p* < 0.001), number of passes (aOR, 1.986; 95% CI, 1.314–3.001; *p* = 0.001), baseline NWU (aOR, 1.179; 95% CI, 1.016–1.369; *p* = 0.030) and baseline ASPECTS (aOR, 0.755; 95% CI, 0.586–0.975; *p =* 0.030) identified as independent risk factors for FR (Supplementary Table S1).

In the early time window subgroup, increasing age (aOR, 1.090; 95% CI: 1.034–1.148; *p* = 0.001) was significantly associated with higher odds of FR in Model A. When perioperative variables were added in Model B, age (aOR, 1.122; 95% CI: 1.053–1.195; *p* < 0.001) remained a strong independent predictor. In addition, an increased number of passes (aOR, 2.307; 95% CI, 1.296–4.104; *p* = 0.004) was significantly associated with increased odds of FR (Supplementary Table S2).

**Figure 3:**
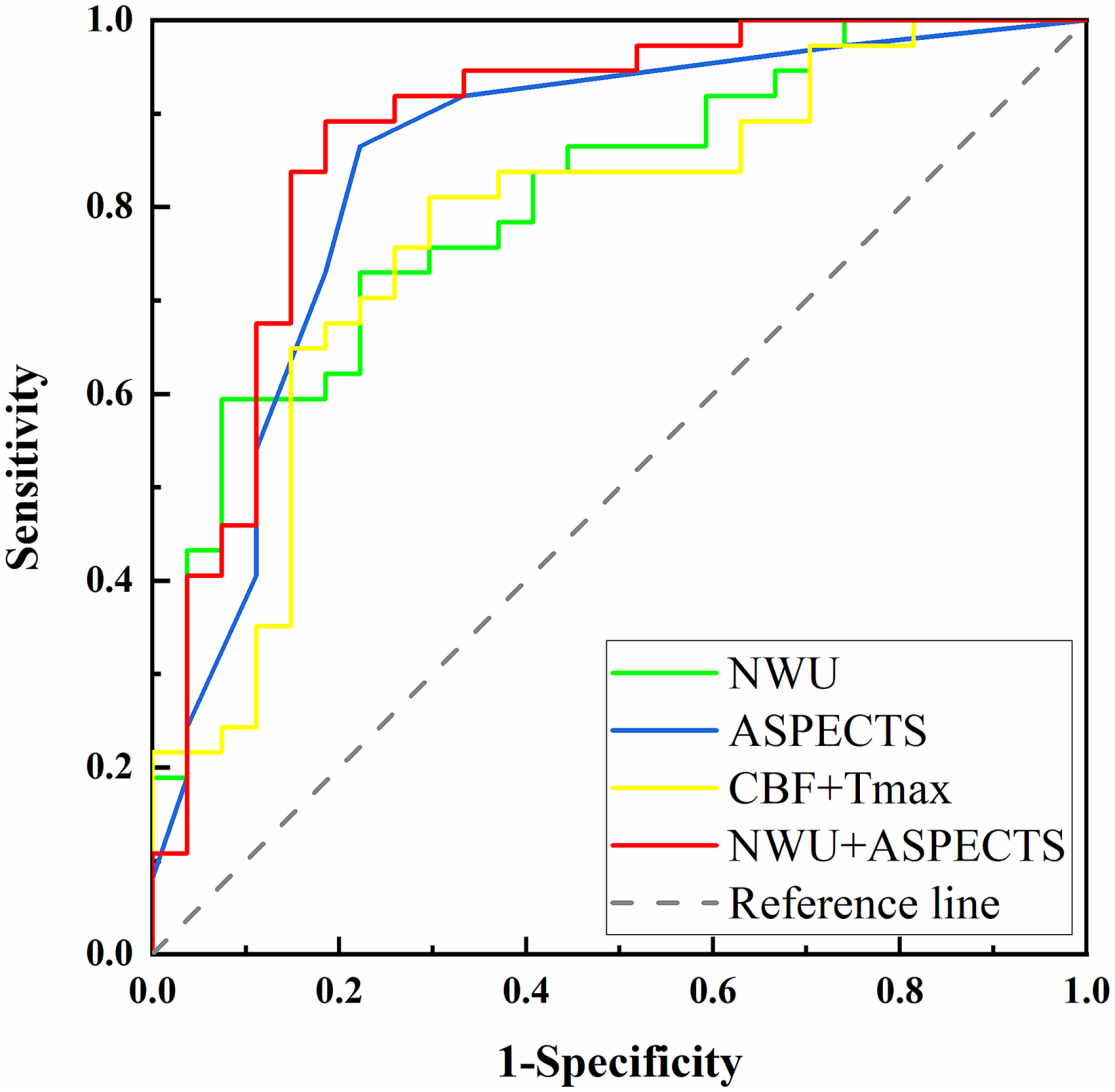
Comparative among different models for predicting FR in the late time window.

**Figure 4:**
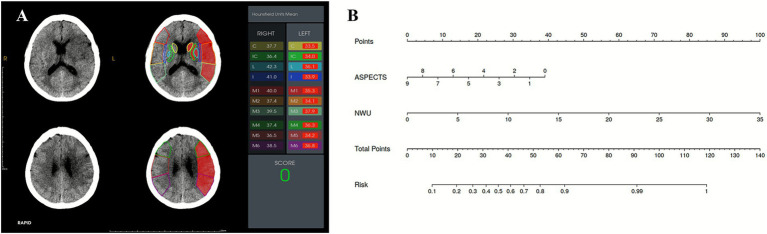
**(A)** A 57-year-old female presented with acute ischemic stroke due to left middle cerebral artery occlusion, imaged 8 h from onset. The ASPECTS was 0, and the NWU based on ASPECTS was 8.95%. **(B)** A nomogram used for predicting FR. According to the ASPECTS (0) and NWU% (8.95%), the probability of FR for this patient at 90 days is 90.44%. The patient experienced FR at 90-day follow-up (mRS: 3).

In the late time window subgroup, baseline ASPECTS and NWU were identified as significant predictors of FR (Table 3). In Model A, lower ASPECTS (aOR, 0.556; 95% CI, 0.340–0.910; *p* = 0.020) and higher NWU (aOR, 1.304; 95% CI, 1.010–1.684; *p* = 0.042) were independently associated with increased odds of FR. After adjusting for perioperative variables in Model B, ASPECTS (aOR, 0.529; 95% CI, 0.307–0.912; *p* = 0.022) and NWU (aOR, 1.373; 95% CI, 1.034–1.823; *p* = 0.028) remained statistically significant. As shown in Table 4 and Figure 3, both NWU and ASPECTS demonstrated good performance in predicting FR. The AUC for NWU was 0.809, achieving 59.5% sensitivity and 92.6% specificity at the optimal cutoff of 9.57%. ASPECTS showed an AUC of 0.846, with 86.5% sensitivity and 77.8% specificity at a cutoff of 6.5. Combining these two parameters increased the AUC to 0.880, with a sensitivity of 89.2% and specificity of 81.5%. Pairwise comparisons of AUC differences revealed no significant disparity among the three models (all *p* > 0.05). The model was evaluated through 5-fold cross-validation, with performance metrics summarized in Supplementary Table S3. Additionally, we have constructed a model using only the CTP indicators Tmax > 6 s and CBF < 30%. This model exhibited an AUC of 0.778, along with a sensitivity of 81.1% and a specificity of 70.4%. The DeLong test demonstrated a significantly higher AUC for the NCCT-based model (ASPECTS+NWU) compared to the CTP-only model (*p* = 0.023). Subsequently, a nomogram was developed to predict FR in the late time window group. Usefully, we validated the nomogram with a case, and the predicted result showed high consistency with the 90-day classification of mRS (Figure 4).

**Table 3:** Logistic regression to predict FR in the late time window.

Variables	Multivariable^A^		Multivariable^B^	
	aOR (95% CI)	*P*	aOR (95% CI)	*P*
Age	1.031 (0.954, 1.115)	0.437	1.031 (0.944, 1.126)	0.500
Sex (Male)	0.252 (0.046, 1.367)	0.110	0.192 (0.029, 1.275)	0.087
NLR	1.091 (0.924, 1.288)	0.306	1.090 (0.918, 1.295)	0.324
Onset to puncture			1.000 (0.999, 1.001)	0.790
Number of passes			2.092 (0.855, 5.116)	0.106
Admission NIHSS	0.948 (0.810, 1.109)	0.503	0.955 (0.803, 1.137)	0.608
Baseline ASPECTS	0.556 (0.340, 0.910)	**0.020**	0.529 (0.307, 0.912)	**0.022**
Baseline NWU%	1.304 (1.010, 1.684)	**0.042**	1.373 (1.034, 1.823)	**0.028**
CBF < 30%	0.980 (0.945, 1.017)	0.281	0.982 (0.942, 1.024)	0.404
HIR	1.012 (0.967, 1.058)	0.609	0.996 (0.945, 1.049)	0.868

^A^excluding perioperative variables. ^B^including perioperative variables; FR, futile recanalization; aOR, adjusted odds ratio; NLR, Neutrophil-to-Lymphocyte Ratio; mRS, modified Rankin Scale; NIHSS, National Institutes of Health Stroke Scale; ASPECTS, Alberta Stroke Program Early CT Score; NWU, net water uptake; Tmax, time to maximum; CBF, cerebral blood flow; HIR, hypoperfusion intensity ratio.

Bold values indicate *P* < 0.05.

**Table 4:** Predication models for FR in the late time window.

Prediction models	Cutoff value	AUC	Sensitivity	Specificity	*P*
NWU (%)	9.57%	0.809	59.5%	92.6%	< 0.001
ASPECTS	6.5	0.846	86.5%	77.8%	< 0.001
NWU + ASPECTS	–	0.880	89.2%	81.5%	< 0.001
CBF + Tmax	–	0.778	81.1%	70.4%	< 0.001

FR, futile recanalization; NWU, net water uptake; ASPECTS, Alberta Stroke Program Early CT Score; CBF, cerebral blood flow; Tmax, time-to-maximum.

## Discussion

This study evaluated the prognostic utility of ASPECTS-based NWU before EVT, a quantitative neuroimaging biomarker, in predicting 90-day functional outcomes among LVO-AIS patients stratified by time window. We found that elevated baseline NWU was associated with higher odds of FR and emerged as an independent predictor of 90-day functional outcomes in late time window patients with an optimal cutoff value of 9.57%. The integration of NWU with the admission ASPECTS into a nomogram provided a robust and clinically practical tool for pre-EVT risk stratification, with an AUC of 0.880 in the late time window cohort.

In this study, 56.55% (95/168) of LVO-AIS patients still experience FR, a rate slightly higher than that reported in previous studies (19, 20). This variance may be explained by several potential contributing factors, including differences in the studied onset-to-admission time windows, varying definitions of successful recanalization, and the enrollment of a cohort comprising patients with larger ischemic cores. Recent evidence from randomized trials has shown that patients with large ischemic infarctions had better functional outcomes with EVT than with medical management alone (21–24). However, other studies have observed that a larger infarct core may reduce the efficacy of EVT (25, 26). The observed differences in results emphasize the necessity of optimizing EVT patient selection criteria in AIS, especially in those with large infarctions.

Extensive cerebral edema development following cerebral ischemia persists as a critical determinant of neurological deterioration (27). The Stroke Treatment Academic Industry Roundtable X has emphasized defining precise neuroimaging biomarkers for cerebral edema, positioning this endeavor as a key priority for adjunctive anti-edema therapeutics (28). Although ASPECTS provided a semi-quantitative evaluation as an inclusion criterion in these trials, the degree of hypoattenuation was not quantified (29). In recent years, NWU has served as a clinically available neuroimaging biomarker based on density measurement that enables quantitative assessment of cerebral edema progression through its dynamic trajectory observed on serial NCCT scans (30). However, this biomarker is susceptible to hemorrhagic transformations and contrast staining after EVT. Reportedly, hyperattenuated intracerebral lesions, including hemorrhagic transformations and contrast staining after mechanical recanalization, were observed in 84.2% of AIS patients (31). Even when overt hyperdense areas were absent on postprocedural imaging, subvisual iodinated contrast staining following endovascular thrombectomy has been demonstrated to significantly impact NWU quantification accuracy, resulting in edema underestimation (32). A previous study has demonstrated that NWU-derived edema indicators failed to predict 90-day functional outcomes in thrombectomy-treated patients with hemorrhagic transformation or EVT history, and NWU application should be strictly constrained to baseline NCCT analysis (33).

Therefore, our research integrates baseline NWU into a multimodal prognostic model to predict FR following successful recanalization. Our key finding is that the predictive value of NWU exhibits a temporally window dependent. We observed that an elevated baseline NWU was associated with FR, regardless of the time window. Multivariable analysis confirmed its role as an independent predictor of FR in patients presenting in the late time window, irrespective of perioperative variables. This is consistent with the evidence of previous studies on stroke onset time using NWU (34, 35). However, in the subsequent early time window subgroup analysis, NWU did not demonstrate independent predictive ability. It is not surprising that interindividual variability in post-stroke edema progression may be associated with the observed nonlinear logarithmic relationship between ASPECTS-NWU measurements and time from symptom onset (36). This result may be due to the patient having mild cerebral edema within 6 h, which reduces the sensitivity of NWU detection. The weak positive correlation between NWU and onset time (*ρ* = 0.20), alongside its moderate association with core volume (*ρ =* 0.54) and HIR (*ρ =* 0.43), underscores that NWU reflects not just time elapsed but more critically, the severity of ischemic pathophysiology. This accounts for its retained independent predictive power in the late window, suggesting a potential advantage over the simple “time clock” model (36).

Current clinical guidelines advocate using CTP-derived penumbral and core assessment to guide intravenous thrombolysis with alteplase and EVT in AIS patients presenting with unknown onset or in the late time window. However, the association of perfusion imaging-guided ischemic lesion analysis with better clinical outcomes in patients with an extended time window continues to be a contentious issue. Current evidence suggests that CTP-derived penumbral/core volume quantification carries risks of overestimating irreversible ischemic damage in AIS, which may inadvertently exclude potential candidates who could derive clinical benefits from EVT (37). The TENSION trial selected patients for EVT based on NCCT-ASPECTS alone and demonstrated treatment effects consistent with trials that relied on advanced imaging modalities (38). Particularly in patients with low ASPECTS, growing evidence highlights the potential of NWU as a selection tool for EVT (39–41). Our study found differences in the infarct core (CBF < 30%) between the FR and the non-FR groups, regardless of the time window. While it provides valuable hemodynamic information, it was not an independent prognostic marker. In contrast, the AUC of ASPECTS and NWU for predicting poor functional outcomes with a late time window in AIS was 0.849 and 0.809, respectively. Notably, both NWU and ASPECTS remained independent predictors of FR in the late time window when ASPECTS was added to the multivariable logistic regression analyses. A significant observation is that the combined NCCT model (ASPECTS + NWU) achieved a statistically superior AUC (0.880) compared to a model based solely on CTP parameters (CBF < 30% + Tmax > 6 s, AUC = 0.778, *p* = 0.023). As NCCT remains the most accessible and rapid imaging modality for AIS evaluation, these findings suggest that quantitative NWU combined with ASPECTS may serve as a viable alternative selection criterion for the late time window AIS patients, particularly benefiting primary stroke centers lacking advanced CTP capabilities. The nomogram we have established can be easily used for family-centered communication, physician decision support, and healthcare resource optimization before EVT.

This study has several limitations. First, the assessment of NWU was derived from ASPECTS-defined anatomical regions, which may not be entirely consistent with the actual ischemic territories. Nevertheless, NWU quantification provides a rapid and objective method for assessing cerebral edema severity, with clinical utility in AIS settings. Second, this was a single-center, retrospective study, with the principal constraint being the lack of an external validation cohort to confirm the generalizability of our findings. Future multicenter investigations incorporating large samples are required to reinforce the robustness of these observations. Third, the exclusion of patients without successful recanalization limits the generalizability of NWU to this population.

In conclusion, LVO-AIS patients with FR exhibited higher NWU values at admission, regardless of the time window. In addition to ASPECTS, quantitative NWU is another independent risk factor for predicting FR in the late time window cohort. Future studies should incorporate NWU as an imaging criterion for treatment decisions. This developed nomogram, which integrates NWU with ASPECTS, represents a clinically practical and noninvasive tool for pre-EVT individualized risk stratification of FR.

## Data Availability

The raw data supporting the conclusions of this article will be made available by the authors, without undue reservation.
