# Supplementary material for: Net water uptake and ASPECTS in predicting futile recanalization for acute large vessel occlusion stroke: insights from time window stratification
Source: Front Neurol. 2026 Feb 4;17:1741637. doi: 10.3389/fneur.2026.1741637 (PMC12913068; doi:10.3389/fneur.2026.1741637)
Supplement: Supplementary file 1 [file Table_1.docx]

Supplementary Material

**Supplementary Table 1. Logistic regression to predict FR in full cohort**

| Variables | Multivariable ^A^ | | Multivariable ^B^ | |
| --- | --- | --- | --- | --- |
|  | aOR (95% CI) | *P* | aOR (95% CI) | *P* |
| Age | 1.078 (1.033, 1.124) | **< 0.001** | 1.091 (1.042, 1.143) | **< 0.001** |
| Sex (Male) | 0.657 (0.288, 1.503) | 0.320 | 0.545 (0.222, 1.339) | 0.186 |
| NLR | 1.068 (0.975, 1.169) | 0.157 | 1.061 (0.969, 1.161) | 0.202 |
| Admission blood glucose | 1.075 (0.907, 1.273) | 0.403 | 1.105 (0.922, 1.324) | 0.279 |
| Number of passes |  |  | 1.986 (1.314, 3.001) | **0.001** |
| Admission mRS | 0.826 (0.373, 1.829) | 0.637 | 0.895 (0.391, 2.047) | 0.792 |
| Admission NIHSS | 1.020 (0.934, 1.114) | 0.664 | 1.011 (0.921, 1.109) | 0.820 |
| Baseline ASPECTS | 0.711 (0.555, 0.907) | **0.006** | 0.755 (0.586, 0.975) | **0.030** |
| Baseline NWU% | 1.107 (0.971, 1.262) | 0.129 | 1.179 (1.016, 1.369) | **0.030** |
| Tmax > 6s | 0.999 (0.996, 1.003) | 0.733 | 0.999 (0.996, 1.003) | 0.710 |
| CBF < 30% | 1.006 (0.992, 1.021) | 0.379 | 1.007 (0.991, 1.023) | 0.387 |
| HIR | 1.006 (0.985, 1.027) | 0.570 | 1.005 (0.983, 1.028) | 0.654 |

^A^, excluding perioperative variables; ^B^, including perioperative variables; FR, futile recanalization; aOR, adjusted odds ratio; NLR, Neutrophil-to-Lymphocyte Ratio; mRS, modified Rankin Scale; NIHSS, National Institutes of Health Stroke Scale; ASPECTS, Alberta Stroke Program Early CT Score; NWU, net water uptake; Tmax, time to maximum; CBF, cerebral blood flow; HIR, hypoperfusion intensity ratio.

**Supplementary Table 2. Logistic regression to predict FR in the early time window**

| Variables | Multivariable ^A^ | | Multivariable ^B^ | |
| --- | --- | --- | --- | --- |
|  | aOR (95% CI) | *P* | aOR (95% CI) | *P* |
| Age | 1.090 (1.034, 1.148) | **0.001** | 1.122 (1.053, 1.195) | **< 0.001** |
| Number of passes |  |  | 2.307 (1.296, 4.104) | **0.004** |
| Admission mRS | 2.014 (0.705, 5.756) | 0.191 | 2.367 (0.798, 7.017) | 0.120 |
| Admission NIHSS | 0.976 (0.879, 1.084) | 0.647 | 0.958 (0.856, 1.072) | 0.452 |
| Baseline ASPECTS | 0.763 (0.565, 1.029) | 0.076 | 0.800 (0.584, 1.096) | 0.165 |
| Baseline NWU% | 0.964 (0.796, 1.167) | 0.706 | 1.039 (0.843, 1.280) | 0.723 |
| Tmax > 6s | 0.999 (0.994, 1.003) | 0.558 | 0.998 (0.994, 1.003) | 0.512 |
| CBF < 30% | 1.021 (0.999, 1.043) | 0.065 | 1.021 (0.997, 1.046) | 0.087 |
| HIR | 1.001 (0.976, 1.0226) | 0.952 | 1.004 (0.978, 1.031) | 0.774 |

^A^, excluding perioperative variables; ^B^, including perioperative variables; FR, futile recanalization; aOR, adjusted odds ratio; mRS, modified Rankin Scale; NIHSS, National Institutes of Health Stroke Scale; ASPECTS, Alberta Stroke Program Early CT Score; NWU, net water uptake; Tmax, time to maximum; CBF, cerebral blood flow; HIR, hypoperfusion intensity ratio.

**Supplementary Table 3. Performance metrics of the model using 5-fold cross-validation**

| Label | AUC (95% CI) | | Sensitivity | | Specificity | | Accuracy | | F1 score | |
| --- | --- | --- | --- | --- | --- | --- | --- | --- | --- | --- |
|  | Train | Test | Train | Test | Train | Test | Train | Test | Train | Test |
| 1-fold | 0.84 (0.72, 0.96) | 1(1, 1) | 0.87 | 1 | 0.76 | 1 | 0.82 | 1 | 0.85 | 1 |
| 2-fold | 0.88(0.77, 0.99) | 0.86 (0.67, 1) | 0.87 | 0.86 | 0.76 | 0.67 | 0.82 | 0.77 | 0.85 | 0.80 |
| 3-fold | 0.92(0.84, 0.99) | 0.73(0.40, 1) | 0.90 | 0.88 | 0.86 | 0.40 | 0.88 | 0.69 | 0.90 | 0.78 |
| 4-fold | 0.89(0.78, 0.99) | 0.85(0.62, 1) | 0.90 | 0.75 | 0.86 | 0.80 | 0.88 | 0.77 | 0.90 | 0.80 |
| 5-fold | 0.87(0.77, 0.98) | 0.97(0.89,1) | 0.83 | 0.71 | 0.82 | 1 | 0.83 | 0.83 | 0.85 | 0.83 |
| Mean | 0.87(0.78, 0.98) | 0.87(0.71, 1) | 0.87 | 0.84 | 0.81 | 0.77 | 0.85 | 0.81 | 0.87 | 0.84 |

AUC, area under the curve.
